# How 3D Printing Is Reshaping Translational Research

**DOI:** 10.3389/fbioe.2021.640611

**Published:** 2021-12-10

**Authors:** Elizabeth A. W. Sigston

**Affiliations:** ^1^ Monash Institute of Medical Engineering, Monash University, Melbourne, VIC, Australia; ^2^ Department of Surgery, School of Clinical Sciences at Monash Health, Monash University Melbourne, Melbourne, VIC, Australia; ^3^ Department of Otolaryngology, Head and Neck Surgery, Monash Health, Melbourne, VIC, Australia

**Keywords:** additive manufacturing, 3D printing, translational research, bioengineering, systems biology, biomedical, design methodology, entrepreneurship

## Abstract

“Translational Research” has traditionally been defined as taking basic scientific findings and developing new diagnostic tools, drugs, devices and treatment options for patients, that are translated into practice, reach the people and populations for whom they are intended and are implemented correctly. The implication is of a unidirectional flow from “the bench to bedside”. The rapidly emergent field of additive manufacturing (3D printing) is contributing to a major shift in translational medical research. This includes the concept of bidirectional or reverse translation, early collaboration between clinicians, bio-engineers and basic scientists, and an increasingly entrepreneurial mindset. This coincides with, and is strongly complemented by, the rise of systems biology. The rapid pace at which this type of translational research can occur brings a variety of potential pitfalls and ethical concerns. Regulation surrounding implantable medical devices is struggling to keep up. 3D printing has opened the way for personalization which can make clinical outcomes hard to assess and risks putting the individual before the community. In some instances, novelty and hype has led to loss of transparency of outcomes with dire consequence. Collaboration with commercial partners has potential for conflict of interest. Nevertheless, 3D printing has dramatically changed the landscape of translational research. With early recognition and management of the potential risks, the benefits of reshaping the approach to translational research are enormous. This impact will extend into many other areas of biomedical research, re-establishing that science is more than a body of research. It is a way of thinking.

## Introduction

“Translational Research” has been defined as taking basic scientific findings and developing new diagnostic tools, drugs, devices and treatment options for patients, “the bench to bedside” goal of biomedical research. (Woolf 2008; van der Laan and Boenink 2015; Sanders 2020). The implication is of unidirectional flow with the aim of seeking how scientific knowledge can be applied in a clinical setting. (Rubio et al., 2010). This century has seen translational research dramatically rise in prominence, largely driven by the recognition that statistically few discoveries in “bench” science have had any material impact on human health or clinical practice with a considerable lag time for those that do. (Balas and Boren 2000; Contopoulos-Ioannidis et al., 2008; Trochim et al., 2011).

Modern healthcare demands for innovative, faster and more personalized solutions have seen the convergence of engineering and biomedical research, leading to emergence of the rapidly growing field of bioengineering, driven to a large extent through the application of additive manufacturing. (Homes 2018). Additive manufacturing, otherwise known as 3D printing was first created in the 1980s. It refers to creating a three-dimensional object from a digital model or blueprint through the printing of materials in successive layers. (Ventola 2014; Fan et al., 2020). This technique enables a focus on functional design, rapid prototype production and individual customization. (Parthasarathy 2014; Ventola 2014; Paul et al., 2018; Fan et al., 2020).

3D printing has not only been instrumental to the development of new fields of study, it has brought together multidisciplinary teams from across the spectrum of engineering, medicine, biomedical research, information technology with other stakeholders, including consumers and commercial funders. The result is reframing of multiple aspects of translational research, ranging from how translational research is defined, to the role of multidisciplinary teams, to tools that better replicate human biology, to the fundamental philosophies that drive it and, ultimately, to the pace at which it occurs.

## Evolving Definition of Translation Research

Appreciating the impact of 3D printing on translational research starts with defining what “translational research” means. The term was originally used sporadically during the 1990s in cancer research to describe research that spanned different types or different disciplines of research, such as basic and clinical research, or immunology and molecular genetics. (Rubio et al., 2010). The turn of the century saw increasing concern from medical scientists and public health policy makers that scientific discoveries were failing to generate any tangible human benefit. (Sung et al., 2003) Even though more scientific discoveries were being achieved and at a faster rate, translation into clinical practice was little better than it was 100 years prior. (Balas and Boren 2000). Studies estimated it took 17–24 years for 14% of new scientific discoveries to enter day-to day clinical practice. (Westfall et al., 2007; Contopoulos-Ioannidis et al., 2008). Lag time and lack of practical impact has ramifications not only for biomedical research, patients and the public but also for governments and funding bodies who are accountable for ensuring resources invested into biomedical research will amount to some measurable improvement in health outcomes. (Woolf 2008; Trochim et al., 2011; Schwartz and Macomber 2017; Sanders 2020).

In June 2000 the initial meeting of the Clinical Research Roundtable of the Institute of Medicine (Sung et al., 2003), a body founded under the charter of the National Academy of Sciences in the United States, was convened to address these concerns. (Fallon 2002). From this arose the concept of ‘translational research’ as the taking of basic scientific findings and developing new diagnostic tools, drugs, devices and treatment options for patients. (Woolf 2008). Obstacles to this progression were defined as “translational blocks” described as T1, the translation of basic science to human studies, and T2, the translation of new knowledge into clinical practice and healthcare decision making. (Sung et al., 2003).

**FIGURE 1:**
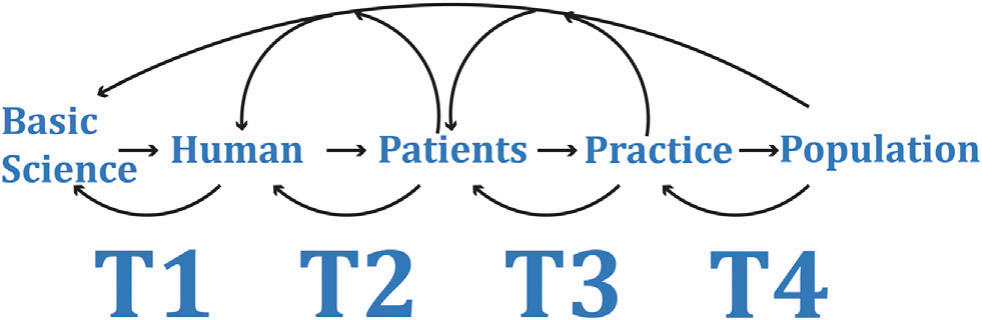
Traditionally translational research was described as the process of taking a basic scientific discovery and working out how that knowledge may be applied at the bedside. The limited progress from discovery to creating an impact on clinical practice and the slow pace at which this occurs has seen an evolution in the way translational research is both defined and approached. Translational research is now considered as a multidirection, cyclic process with starting point being any of the translational phases T1 to T4. Phase T1 represents translation of basic science to application in humans, T2 from human application to patients, T3 from patients into accepted clinical practice and T4 from clinical practice to the population. Each phase can move in either direction and can feedback or feed forward to influence or direct the other phases.

Over time the “T” has changed from representing a translational block to representing a translational phase. Currently there is general consensus on the definitions of T1 through to T4 (Figure 1). (Fort et al., 2017) Additionally, T0 has been proposed to represent genomic-wide association studies and basic science discovery. (Gannon 2014; Fort et al., 2017). T5 is used in some forums to represent international adoption of a clinical practice. (Fort et al., 2017).

The other major shift has been acceptance that translational research needs to be multidirectional, recognizing that data and observations from clinical practice, individual and collective behaviours are critical in creating real world impacts. (Woolf 2008; Cohrs et al., 2015; van der Laan and Boenink 2015; Jia 2016; Smith et al., 2017). The European Society for Translational Medicine (EUSTM) defines translational research as *an interdisciplinary branch of the biomedical field supported by three main pillars: benchside, bedside and community.* (Cohrs et al., 2015). Merging this concept with the translational phases, modern translational biomedical research can be viewed as a multidirectional integrated process (Figure 1).

## From Pondering to Problem Solving

Biomedical research has been dominated by basic research (Sung et al., 2003; Woolf 2008), that is, research that results in adding to general knowledge and understanding of nature and its laws, but without the practical ends in mind. (Rubio et al., 2010; Patel and Mehta 2016). It follows the fundamental steps of scientific method: observation, hypothesis, experimentation and generalization, favouring a quantitative and analytical approach. (Greenman et al., 2007). Translational research in this methodology, the “bench to bedside” approach, requires working out how that knowledge is then applicable to clinical health scenarios. The implication is of unidirectional flow underpinned by the reductionist philosophy that biology can be explained by breaking it down to chemical or molecular reactions. By simply tying this knowledge together, answers to all clinical questions can be found. As discussed above, translation of biomedical research into clinical practice using this approach has been slow and largely ineffective. (Balas and Boren 2000; Balas and Boren 2000; Westfall et al., 2007; Contopoulos-Ioannidis et al., 2008; Trochim et al., 2011).

In contrast, the engineering method (engineering design) is a systematic approach to finding a solution to a problem. The starting point is identifying and researching the problem, followed by ideation of a solution, planning, development of proof of concept and/or prototyping, testing and re-iterations, then implementation. (Lasser 2013; Greene et al., 2017). The engineering design method compared to traditional biomedical research, deals primarily with something that doesn’t yet exist. (Patel and Mehta 2016).

**FIGURE 2:**
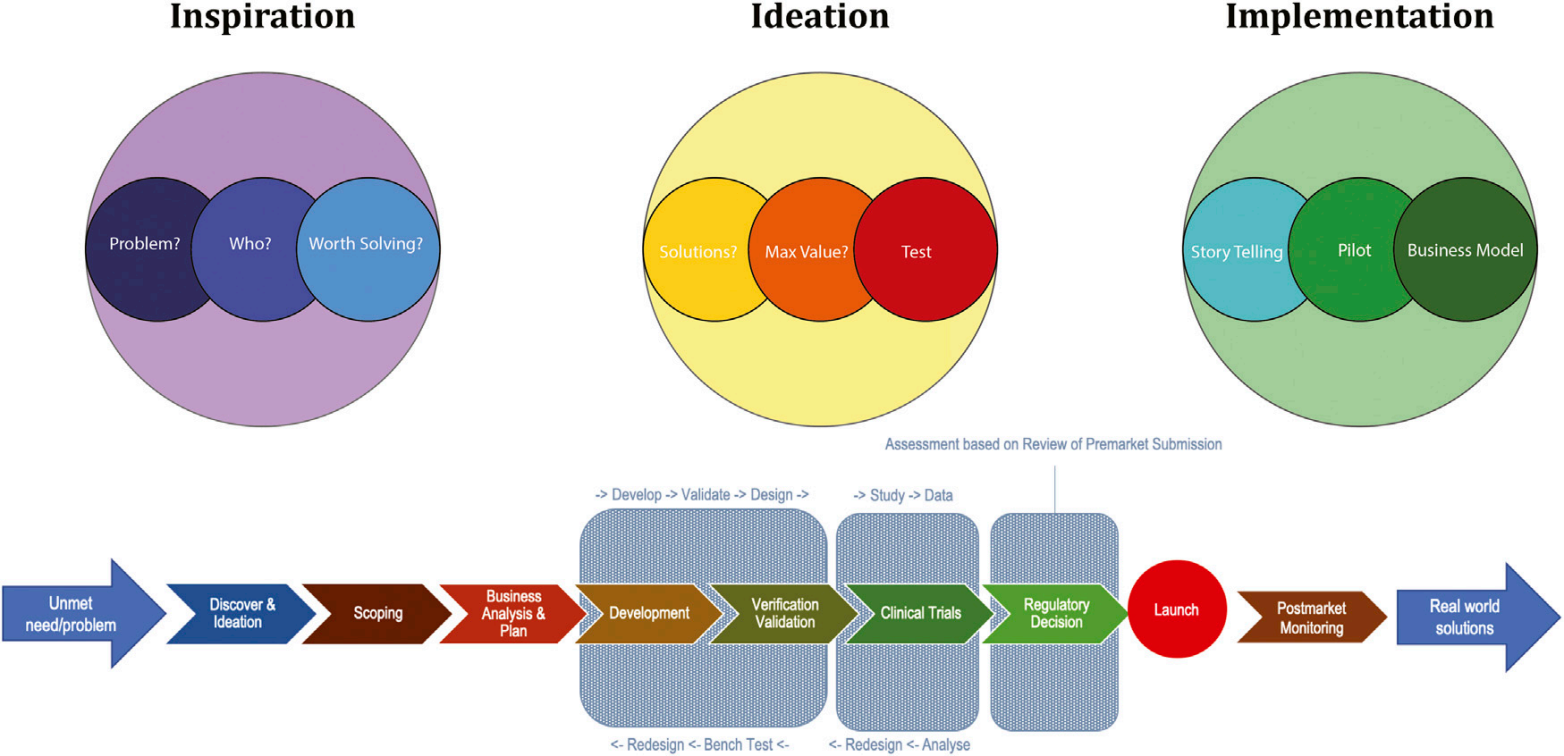
Applying engineering design thinking and entrepreneurial thinking in the translational research process creates a systematic approach to finding a solution to a problem that is worth solving. In biomedical research this starts with identifying an unmet healthcare need or clinical problem. Scoping of the problem and undertaking a business analysis occurs at the beginning, ideas of how the problem may be solved are explored, re-iterated and then progress to implementation and a real world solution. (Flow chart component developed by Monash Institute of Medical Engineering, Monash University.)

The application of 3D technologies to human biology and medicine in order to improve healthcare and healthcare outcomes has contributed to the rise of the new field of biomedical engineering. (Moffat 2017). Use of problem solving engineering design methodology in biomedical research places an unmet clinical or healthcare need as the problem to be solved, driving translational research in a targeted direction. (Figure 2). For example, assessing the clinical problem of high rates of plate extrusion, screw loosening and/or poor osseointegration in mandibular reconstructions in head and neck cancer patients (Shaw et al., 2004; Goh et al., 2008) and finding poor match in elastic properties of titanium plates to native bone produces stress shielding (Gutwald et al., 2017; Soro et al., 2019), enabled development of an alternative alloy produced through 3D printing with better mechanical properties and improved osteogenic potential. (Soro et al., 2019; Brodie et al., 2021).

## A Systems Approach

Complex systems exist everywhere in nature, including the human body. (Van Regenmortel 2004; Mazzocchi 2008; Mesarovic, 2017). They are dynamic and have the ability to self-correct through cyclic feedback loops. A systems approach considers a complex system as a whole and involves understanding interactions and influences between various components in a system to solve complex problems. (Patel and Mehta 2016). At the core are the concepts of emergence and interrelatedness, that a system is more than the sum of its parts. (Patel and Mehta 2016; Greene et al., 2017; Kolodkin, 2017).

Ironically, whilst the engineering field adopted systems thinking generating the fields of cybernetics and system dynamics (Greene et al., 2017), biomedical research followed the reductionist path of molecular biology, identifying the gene as the fundamental unit of biological information and chemistry the effective mechanistic explanation of biological processes. (Nurse 2008; Green 2017). Though this approach has led to significant improvement in understanding of human disease, translation to clinical impact has not been fast or frequent. Increasingly gaps and paradoxes arising from this assumption (Sonnenschein and Soto 2008; Bertolaso et al., 2011; Baker 2012; Bizzarri and Cucina 2014; Bertolaso, 2017) has led to acknowledgement that biological complexity has been overlooked (Nurse 2008) resulting in the rise of “systems biology”, a term that describes the quantitative analysis of the dynamic interactions among several components of a biological system with the aim to understand the behavior of the system as a whole.

Systems thinking allows biomedical research and engineering to dovetail into translatable solutions (Chien et al., 2015) and 3D printing is at the heart of it. Two-dimensional monolayer cells cultures do not reflect biological complexity. The importance of 3D culture was initially highlighted by Mina Bissell and her team, demonstrating that both structural and biochemical cues are required for mammary acinar development. (Schmeichel et al., 1998). This led to development of “organoids”, miniature organs derived from tissue-resident stem/progenitor cells or embryonic stem cells in the presence of organ-specific cues and matrices in culture dishes. Organoids resemble an organ in both structure and function. (Rawal et al., 2021). 3D printing is now being used to reliability reproduce organoids, tumoroids, and even whole organs that better represent human systems for use in disease and regenerative research with ultimate potential to produce transplantable tissues. (Reid et al., 2018; Mansilla et al., 2021; Rawal et al., 2021).

## Rise of the Prosumer

Availability of 3D printers to the wider public has seen the rise of the “prosumer”, a person who is involved in the co-creation and innovation of the product they use. (Rayna et al., 2015). In healthcare, prothesis have been the target of prosumers. e-NABLE, an online global community of volunteers is an example of prosumers not waiting for companies or governments to find solutions. The open-source designs created by e-NABLE volunteers allow their community to use personal 3D printers to help those born with missing fingers and hands or who have lost them due to war, natural disaster, illness or accidents. Over 8,000 recipients have been helped. (eNABLE).

The Covid-19 pandemic has also driven prosumers to the fore with 3D printing used widely to fill the need for low-cost, rapid fabrication of medical devices and personal protective equipment as the world faced a short fall from more established production lines. Frontline healthcare workers became actively involved in designing items for personal and peer use. Not only was 3D printing used to crucially help with this shortfall, it demonstrated, in real time, how fast translation can be when problem and outcome focused. (Radfar et al., 2021).

Public awareness of the ability to use 3D printing to customize prothesis, implants and other devices is increasing demand for such products. In turn, this drives innovation and translation. There has been an explosion in prosumer driven custom-made prosthetics, such as Free 3D Hands and Art4Leg, allowing patients to be involved in designing their own limb or casts with an increasing focus on developing better functionality at lower cost. (Ventola 2014; Nawrat 2018; Paul et al., 2018; Aimar et al., 2019). Surgeons commonly use 3D models and templates to plan and improve surgery, resulting in shorter operating times and better functional outcomes. (Shinomiya et al., 2018; Witjes et al., 2018; Aimar et al., 2019; Fan et al., 2020).

## Entrepreneurialism

Entrepreneurialism seeks creation or extraction of value through creativity and innovation. (Patel and Mehta 2016). The historic lack of return on biomedical research is a prime driving force behind the increased need for entrepreneurial thinking in translational research to continue to attract funding and investment. (Woolf 2008; Molas-Gallart et al., 2016). 3D printing has done that partly because the products are physically tangible and immediate. The healthcare impacts and hence return on investment is visible. A burgeoning industry based on 3D printing has evolved. Established medical device companies, such as Stryker and Medtronic, are investing heavily in 3D printing, for customized implants, training and simulation, and to reduce development time via the use of rapid prototyping. (Medtronic 2017; Sher 2020). Multiple new companies have arisen producing 3D printers, 3D printing material and digital files, creating such products as “organs-on-a-chip”. (Jiang et al., 2017; Paul et al., 2018; Aimar et al., 2019; Fan et al., 2020; Wu et al., 2020).

The impact is that the foundations of good business (creating or delivering something of value that people want or need, at a price they are willing to pay, in a way that meets their needs and expectations and that will generate enough profit to make it worthwhile for the owners to continue operations) (Kaufman 2013), need to be worked into the translational research design. Increasingly business management strategies are being employed in translational research to improve efficiency and cost-effectiveness. (Schweikhart and Dembe 2009).

## Obstacles and Pitfalls

3D printing is an exciting technology. It fires the imagination, bringing with it the biggest risk: exposing scientific research to public hype. The Gartner Hype Cycle is a graphic representation of the maturity and adoption of technologies and applications, and how they are potentially relevant to solving real world problems. (Gartner 2020). Whilst publicity can be good, hype can inflate public expectations and erode trust, undermining the scientific process and profession. (Rinaldi 2012). In healthcare, public trust is paramount for uptake of new ideas and technologies. They need to be seen to deliver on their promise.

Complex regulatory requirements are seen as a major barrier. (Fudge et al., 2016). New requirements for medical devices introduced in Europe in 2017 and subsequently by other jurisdictions to improve the safety of medically implanted devices (European Parliament and Council on medical devices 2017) followed a significant breast implant issue. (Russell 2017). Whilst appropriate for mass production implants they lack the nuance required for more customized 3D printed products. Legislation for 3D printing of devices in many jurisdictions doesn’t distinguish between difference purposes. A 3D model for surgical planning or educating patients can fall into the same regulation as devices for implant. Use of 3D printing across many facets of translational research makes it difficult to produce cohesive regulation. (Christensen and Rybicki 2017). Achieving balance of safety and social responsibility without generating too much stifling red tape is challenging. (Christensen and Rybicki 2017; Adamo et al., 2018; Aimar et al., 2019).

3D printing highlights the need for increased and early multidisciplinary collaboration in translational research. (Fudge et al., 2016; Homes 2018). Whilst a major benefit in ensuring research is directed to a real world need, lack of clear definition of concepts and different language between engineers, clinicians and other stakeholders can create confusion, lack of clarity and direction that can hamper progress. (Woolf 2008; LeClair et al., 2020). Competing demands on clinicians time with lack of protected and funded time for research impedes stakeholder engagement. (LeClair et al., 2020). The application of engineering principles to human biology and medicine in order to improve healthcare and healthcare outcomes brings with it the burden of ethical responsibilities to bioengineers to anticipate the consequences of their technological designs for medical practice in a manner similar to a medical practitioner. These include do no harm, informed consent, confidentiality and dignity. Tissue engineering, use of biomaterials and implants, and neural engineering each generate specific numerous ethical concerns that will need to be addressed. (Moffat 2017).

Commercial partnerships and funding arrangements can generate conflict of interest and pitfalls, through looking for faster, more expedient ways to bring devices or technologies to market, or creating prestige to advance further funding opportunities without paying attention to the way this is achieved. (Molas-Gallart et al., 2016). The disastrous artificial tracheal implant saga highlights this can occur at even the most respected institutes. (Schneider 2016). Repercussions impact not only the individuals and institute involved but undermine public confidence across the medical implant device industry and potentially the view taken by regulators.

Finally, measuring and evaluating progress is unstandardized. Personalization makes outcome measures harder to determine and standardize and risks putting the need of the individual before the community. Many academic organizations reward work based on individual output primarily through publications and grants, rather than team outputs, patents, trade secrets, and impact on health outcomes. This can dissuade collaboration and translation.(Fudge et al., 2016; Smith et al., 2017; Clay et al., 2019).

## Future

3D printing has demonstrated that when healthcare needs, such as prosthetic limbs, are the driver, real world outcomes can be achieved at a faster pace with less waste and lower costs. As the cost of 3D printers and materials reduce, these technologies will become more widely available. Use of 3D printed organoids is already seeing the cost of pharmaceutical development being reduced and has potential to reduce, if not eliminate, use of animal experimentation. (Fan et al., 2020; Rawal et al., 2021). Prosumer groups, such as e-NABLE, have demonstrated that it will not necessarily be the wealthiest countries to benefit.

The temptation may be to either overregulate or forgo proper safety assessment. Jurisdictions that are agile in adapting their regulations to ensure a balance of safety whilst not stifling progress will be the big winners.

Those countries or groups who can connect and engage with the end-users, build functioning multi-disciplinary teams across a myriad of disciplines, and maintain focus on meeting the desired healthcare outcomes will achieve faster translation and better return on research, government and commercial funding. Finally, those who are able to grasp how 3D printing technologies can be used in understanding complex systems will be the ones to tap into the wealth of knowledge that has yet to produce healthcare impact.

## Summary

In this century, 3D printing has moved from the realm of fiction to generating impact on health outcomes and healthcare across the spectrum. 3D printing has been pivotal in the merging of engineering and biomedical fields. In this way, it has helped shape how translational research is defined, understood and pursued.

## Data Availability

The original contributions presented in the study are included in the article/Supplementary Material, further inquiries can be directed to the corresponding author.
